# Novel Insights Into the Struggle Against Biofilm: The *Psy* Omp38 Protein From the Antarctic Marine Bacterium *Psychrobacter* sp. TAE2020

**DOI:** 10.1111/1751-7915.70249

**Published:** 2025-10-07

**Authors:** Diana Olimpo, Caterina D'Angelo, Paola Imbimbo, Marco Morelli, Maria Luisa Tutino, Andrea Carpentieri, Daria Maria Monti, Eugenio Notomista, Ermenegilda Parrilli

**Affiliations:** ^1^ Department of Chemical Sciences University of Naples “Federico II” Naples Italy; ^2^ Department of Biology University of Naples “Federico II” Naples Italy

**Keywords:** antibiofilm, cold‐adapted bacteria, outer membrane protein

## Abstract

Antibiofilm molecules can enhance the effectiveness of antibiotics and prevent biofilm formation. Antarctic marine bacteria have been found to secrete antibiofilm molecules, likely as part of a strategy for competitive survival. The protein‐polysaccharide complex CATASAN, produced by the Antarctic bacterium *Psychrobacter* sp. TAE2020, has been shown to interfere with all stages of *Staphylococcus epidermidis* biofilm development. This study investigates the contribution of *Psy*Omp38, the protein component of CATASAN, to the complex's antibiofilm activity. The protein was heterologously expressed in *Escherichia coli* , purified, and characterised, revealing its ability to inhibit *Staphylococcus epidermidis* adhesion to surfaces, interfere with biofilm formation, and disrupt mature biofilms. Following biocompatibility assessment, *Psy*Omp38 was tested in combination with vancomycin as a potential treatment for established infections, revealing a reduction in the minimum biofilm eradication concentration (MBEC) of vancomycin. The potential of *Psy*Omp38 for material functionalisation was also explored. The protein was deposited onto silicone‐based surfaces, and the coated materials were tested in a continuous‐flow system that simulated real‐life conditions. Additionally, the three‐dimensional structure of *Psy*Omp38 was predicted and compared with homologous proteins. The structural analysis not only revealed the unique features of *Psy*Omp38 but also provided important insights into the molecular mechanisms underlying its antibiofilm activity.

## Graphical abstract

The protein *Psy*Omp38 inhibits biofilm formation and disrupts the mature biofilm of *Staphylococcus epidermidis* . The use of this antibiofilm protein in combination with vancomycin increases the efficacy of the antibiotic, reducing its MBEC on *S. epidermidis* . Furthermore, PDMS coated with *Psy*Omp38 prevents *S. epidermidis* biofilm, suggesting its application in the development of antibiofilm materials aimed at preventing the onset of biofilm‐associated infections.

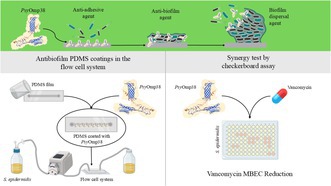

## 1 Introduction

Biofilms are dynamic, self‐assembled microbial communities embedded in an extracellular polymeric matrix (Flemming et al. 2016). They provide stability, enhance nutrient capture, and protect microorganisms from environmental stressors, with their extracellular polymeric substances (EPS) acting as a barrier against external threats such as antimicrobials (Flemming and Wingender 2010). As a result, bacteria within biofilms exhibit resistance levels 10‐ to 1000‐fold higher than their planktonic counterparts, contributing significantly to the global antimicrobial resistance crisis (Muhammad et al. 2020). This crisis is responsible for millions of deaths annually and imposes a huge economic burden. To combat this challenge, novel strategies are needed to enhance antimicrobial efficacy while ensuring biocompatibility, environmental sustainability, cost‐effectiveness, and long‐term stability (Bi et al. 2021; Murray et al. 2022). One promising approach involves the use of antibiofilm molecules to boost the effectiveness of traditional antibiotics and prevent biofilm formation by pathogenic strains (Bi et al. 2021; Abdelhamid and Yousef 2023). To fully harness the potential of these molecules, it is essential to underline that microbial biofilm establishment is a complicated, multi‐step process comprising three distinct phases: adhesion, maturation, and dispersion (Flemming et al. 2016). Antibiofilm molecules can interfere with any of these stages by preventing surface adhesion, disrupting biofilm development, or promoting the dispersion of mature biofilms. Given the wide range of potential molecular targets, it is unsurprising that antibiofilm compounds exhibit significant chemical and structural diversity, encompassing proteins, enzymes, and quorum‐sensing inhibitors (Abdelhamid and Yousef 2023).

The search for new antibiofilm agents has led researchers to extreme environments such as Antarctica, an untapped reservoir of microbial biodiversity with potential sources of antibiofilm molecules (Núñez‐Montero and Barrientos 2018). Since biofilm formation is a survival strategy in highly oligotrophic environments, producing antibiofilm compounds may provide a competitive advantage by inhibiting competing microorganisms. Indeed, recent studies have reported the secretion of antibiofilm molecules by marine Antarctic bacteria (Parrilli et al. 2015; Artini et al. 2019, 2023; Riccardi et al. 2021).

Several investigations have supported that the cell‐free supernatants of four Antarctic marine bacterial strains, *Pseudoalteromonas haloplanktis* TAC125, *Psychrobacter* sp. TAE2020, *Pseudomonas* sp. TAE6080, and *P. haloplanktis* TAD2020 effectively stop biofilm development and boost the dispersion of mature biofilms in ESKAPE pathogens (Artini et al. 2023). Additionally, marine cold‐adapted bacteria have been found to avoid biofilm development in other pathogens, such as *Staphylococcus epidermidis* (Casillo et al. 2017; D’Angelo et al. 2024; Papa et al. 2013). A recent study identified a molecule, CATASAN (D'Angelo et al. 2022), produced by the marine bacterium *Psychrobacter* sp. TAE2020, which interferes with multiple stages of *S. epidermidis* biofilm development. CATASAN is a complex composed of a protein (*Psy*Omp38), lipopolysaccharide, and a high‐molecular‐weight polysaccharide. *Psy*Omp38 is homologous to certain outer membrane proteins of the OmpA family (Sugawara and Nikaido 2012; Zhou et al. 2023), frequently found in Gram‐negative bacteria. *Psychrobacter* sp. TAE2020 Omp38 reveals 46% identity with AlnA from *A. radioresistens* K53, a key element of the emulsifying complex Alasan (Pessione et al. 2003; Toren et al. 2003; Walzer et al. 2006). Other OmpA‐like proteins from *Acinetobacter*, such as *Acinetobacter* sp. strain ADP1, possess emulsifying ability (Walzer et al. 2006). The extracellular secretion of the OmpA‐like proteins, which possess emulsifying activity, suggests a functional role in increasing the availability of hydrophobic substrates of nutritional relevance. Furthermore, OmpA has been implicated in the regulation of OMV biogenesis, contributing to bacterial adaptation and being under stress conditions (Schwechheimer and Kuehn 2015). In *A. baumannii* strains ATCC 17978 and ATCC 19606, respectively (Camarena et al. 2010), the expression of OmpA is also modulated by various environmental factors; for example, ethanol exposure and iron supplementation, while in *A. radioresistens* S13 the presence of OmpA was greater in cells stressed by the presence of phenol or benzoate (Pessione et al. 2003; Toren et al. 2003). In summary, it is evident that outer membrane proteins play several biological roles, and in this study, we focused on investigating the specific contribution of *Psy*Omp38 to the overall antibiofilm activity of CATASAN. To this end, *Psy*Omp38 was heterologously produced in *E. coli* , subsequently purified, and a functional and structural investigation of this protein was performed to uncover its properties. The results revealed that the protein inhibits the adhesion of *Staphylococcus epidermidis* to surfaces, can interfere with biofilm formation, and effectively disrupts mature biofilms. Through a combination of molecular, microbiological, and material‐based assays, we demonstrate that *Psy*Omp38 is not only central to biofilm interference but also holds strong potential for therapeutic and surface‐engineering applications.

## 2 Materials and Methods

### 2.1 Bacterial Strains, Media, and Plasmids

The strains, plasmids, and oligonucleotides used in this study are listed in Table S1. *Psychrobacter* sp. TAE2020 was grown in synthetic medium Gut (Riccardi et al. 2022) at 15°C under vigorous agitation (250 rpm) for 72 h. *S. epidermidis* O‐47 (Raue et al. 2020) and *S. epidermidis* RP62A (ATCC collection no. 3598) were grown at 37°C in BHI (Oxoid, UK); biofilm formation was assessed in static conditions, while planktonic cultures were performed under agitation (180 rpm). For the checkerboard assay, *S. epidermidis* RP62A was grown in cation‐adjusted Muller‐Hinton broth. *E. coli* Top10 strain was used for cloning purposes, while *E. coli* BL21(DE3) and *E. coli* C41(DE3) were used for recombinant protein production. *E. coli* strains were grown in Luria‐Bertani (LB) at 37°C under vigorous agitation at 220 rpm. When required, 50 μg/mL of kanamycin (Sigma‐Aldrich) was supplemented to the medium. All strains were maintained at −80°C in cryovials with 20% glycerol.

### 2.2 Construction of the Expression Plasmids

*Psychrobacter* sp. TAE2020 gene (WP_216581271) was amplified using primers with *Nco*I and *Bam*HI restriction sites (Table S1). The PCR reaction was performed in a volume of 50 μL following the manufacturer's instructions (New England Biolabs, Hitchin, UK). The PCR amplified fragments, 1184 bp (Omp38 + SP) and 1118 bp (Omp38‐SP), were double‐digested with *Nco*I/*BamH*I restriction enzymes, cloned into the vector pET28a previously digested with the same restriction enzymes, and transformed into *E. coli* Top10. The DNA amplified fragments were checked by sequencing.

### 2.3 Production of Omp38 + SP and Omp38‐SP Recombinant Proteins

The pET28a‐Omp38+PS or pET28a‐Omp38‐PS constructs were transformed into *E. coli* BL21(DE3) or C41(DE3) competent cells, plated on LB‐agar supplemented with 50 μg/mL kanamycin (Sigma‐Aldrich, Steinheim, Germany), and incubated overnight at 37°C. The transformed cells were then used for protein expression. Briefly, individual transformed colonies were inoculated into 3 mL of LB medium containing 50 μg/mL kanamycin and incubated at 37°C with shaking at 220 rpm. After 24 h, the pre‐culture was diluted into 50 mL of fresh LB medium with 50 μg/mL kanamycin to an initial OD_600nm_ of 0.1 and grown at 37°C, 220 rpm. When the culture reached an OD_600nm_ of 0.4, the temperature was shifted to 25°C. Upon reaching an OD_600nm_ of 0.6, protein expression was induced with 1 mM IPTG for Omp38 + SP and 2 mM IPTG for Omp38‐SP. In both cases, 1 mM CaCl_2_ was added at the time of induction. Cultures expressing Omp38 + SP were incubated for 24 h at 20°C, whereas those expressing Omp38‐SP were incubated for 6 h at 25°C. To evaluate protein production, cells were harvested by centrifugation at 6500× *g* for 1 h at 4°C, and resuspended in lysis buffer (50 mM TrisHCl, 500 mM NaCl, EDTA‐free protease inhibitor (Roche‐tablets), 0.1 mg/mL lysozyme, and H_2_O). Cell lysis was performed by sonication using a Bandelin SONOPULS ultrasonic homogeniser equipped with an M72 probe (suitable for 2 mL to 30 mL) at 35% amplitude for 30 min, alternating 30‐s on/off cycles. The obtained lysates were centrifuged (7000× *g* for 30 min at 4°C) to separate the soluble (sol) and insoluble (insol) fractions.

### 2.4 Denaturation‐Renaturation Protocol

The insoluble fractions were denatured following McDonnell et al., with slight modification (McConnell and Pachón 2011). The inclusion bodies were incubated in Denaturation Buffer at room temperature (RT) in dynamic conditions. After the incubation, the remaining insoluble material was removed by centrifugation at 6500× *g* for 1 h at 4°C. For refolding experiments, samples were dialysed against 50 mM Tris‐HCl pH 9.0 using a 12 kDa dialysis tube (Sigma‐Aldrich).

### 2.5 Proteins Purification

The isoelectric points (pI) of Omp38 + SP and Omp38‐SP are 4.53 and 4.46, respectively (Expasy tool analysis). The proteins were renatured in 50 mM TrisHCl at pH 9.0, rendering the proteins negatively charged and suitable for binding to an anion exchange column. Q Sepharose Fast Flow resin (Cytiva) was used, with 4 mL of resin loaded with 5 mg of protein. The column was equilibrated with H_2_O 10 C.V, elution buffer 10 C.V, and binding buffer 10 C.V. The renatured samples (RN) were loaded for 2 h at RT in dynamic conditions. Elution was performed using a NaCl gradient.

### 2.6 SDS‐PAGE Analysis

To analyse the protein profile by SDS‐PAGE, bacterial pellets corresponding to approximately 1 OD unit were collected at the end of growth by centrifugation at 13,000× *g* for 20 min at 4°C and resuspended in 60 μL of Laemmli buffer. The total cell extracts were then boiled at 95°C for 20 min, rapidly cooled on ice for 1 min, and centrifuged at 13,000× *g* for 30 s at RT. For the solubility analysis of Omp38 proteins, 30 μL of either the soluble (sol) or insoluble (insol) fractions were recovered and mixed with 10 μL of Laemmli buffer. The samples were then boiled at 95°C for different durations (5 min for sol and 10 min for insol), rapidly cooled on ice for 1 min, and centrifuged at 13,000× *g* for 30 s at RT. To assess protein renaturation, 30 μL of denatured (DN) and renatured (RN) fractions were prepared and analysed on SDS‐PAGE after boiling for 5 min in Laemmli buffer. To evaluate purification efficiency, 30 μL of flow‐through (FT), wash (W), and elution fractions (E25%: 50 mM TrisHCl +250 mM NaCl; E100%: 50 mM TrisHCl +1 M NaCl) were mixed with 10 μL of Laemmli buffer. SDS‐PAGE gels were stained with Coomassie Brilliant Blue, and protein molecular weights were estimated by comparison to a molecular mass standard (Precision Plus Protein Prestained Standards—Dual Colour, Bio‐Rad).

### 2.7 UREA‐PAGE Analysis

Protein denaturation and separation were performed using a 10% urea–polyacrylamide gel electrophoresis (UREA‐PAGE) following Salzano et al. Protocol (Salzano et al. 2013). The resolving gel was prepared with 8 M urea in a final volume of 10 mL, consisting of 3.3 mL of 30% acrylamide/bisacrylamide solution, 1 mL of 10× TBE buffer, 4.8 g of urea, and deionised water to reach the final volume. Protein samples were mixed in a 1:1 ratio with 2× urea sample buffer, composed of 3.6 g urea, 1 mL of 1 M TrisHCl (pH 6.8), 2 mL glycerol, 0.1 M DTT, 10 μL of 0.05% bromophenol blue, and deionised water to a final volume of 10 mL. Samples were denatured by heating at 90°C for 5–10 min before loading.

### 2.8 Confocal Laser Scanning Microscopy

The antibiofilm activity of Omp38 proteins against *S. epidermidis* was assessed as previously reported, with minor modifications (D’Angelo et al. 2024). Cultures were incubated for 24 h at 37°C in the presence or absence (control) of Omp38 proteins (10 μg/mL). Biofilm viability was evaluated using the Filmtracer LIVE/DEAD kit according to the manufacturer's protocol. After washing with sterile PBS, CLSM imaging was performed using excitation/emission wavelengths of 480/500 nm and a 20× NA 0.8 objective. Z‐stacks were acquired with 1‐μm steps to cover the entire biofilm depth. Images were processed with ZEN Black 3.0 and analysed quantitatively using COMSTAT software (Heydorn et al. 2000) to measure biomass, average thickness, and roughness coefficient (Ra*). Two independent biofilm replicates were analysed per condition. For preformed biofilms, *S. epidermidis* cultures (300 μL, OD_600_ 0.1 for RP62A and 0.001 for O‐47) were incubated for 24 h, washed, and then treated with Omp38 proteins (100 μg/mL) or buffer control for an additional 24 h. CLSM imaging and analysis were conducted as described above.

### 2.9 Situ Hydrolysis, LC–MS/MS Analysis and Protein Identification

Trypsin digestion was conducted after excising protein bands from the SDS‐PAGE gels according to the protocol described in Xing et al. (2020). Proteins were analysed on an LTQ Orbitrap XL Hybrid Ion Trap‐Orbitrap MS System (Thermo Scientific, Bremen, Germany) according to the previously reported methods (Cipolletta et al. 2024). The acquired MS/MS spectra were converted to Mascot Generic files (.mgf) format and subsequently queried against a customised database TAE2020 using a licensed version of Mascot software (version 2.4.0) available at www.matrixscience.com for protein identification, following the parameters previously used (Cipolletta et al. 2024).

### 2.10 Biofilm Inhibition Assay

Biofilm formation was assessed as previously described (D'Angelo et al. 2024) with minor modifications. To investigate the impact of Omp38 proteins on biofilm development, sterile 96‐well flat‐bottom polystyrene plates were inoculated with *Staphylococcus epidermidis* RP62A or O‐47 (OD_600_ ~0.1 and 0.001, respectively) in BHI medium. Omp38 proteins were added at different concentrations, while TrisHCl buffer (50 mM, pH 9.0) served as a control. Plates were incubated aerobically at 37°C for 24 h. Biofilm biomass was quantified by crystal violet staining, and the dye bound to adherent cells was solubilised in 20% (*v*/*v*) glacial acetic acid and 80% (*v*/*v*) ethanol before measuring absorbance at 590 nm using a microplate reader (Benchmark Plus Microplate Spectrophotometer). The effect on preformed biofilms was evaluated by first allowing biofilms to develop for 24 h under the same conditions. After removing non‐adherent cells by PBS washing, Omp38 proteins or buffer controls were added and incubated for an additional 24 h. Biomass quantification was performed as described above. All conditions were tested in six technical replicates and repeated at least twice in independent experiments.

### 2.11 Surface Coating Assay

The antiadhesive activity was assessed as described in D'Angelo et al. (2022). After depositing and drying the protein sample at a concentration of 10 μg/mL on the surface, *S. epidermidis* was added, and biofilm formation was assessed by crystal violet staining. The presence of a clear area at the deposition site indicates inhibition of adhesion.

### 2.12 Synergy Testing by Checkerboard Assay

The combined activity of vancomycin and *Psy*Omp38 protein against *S. epidermidis* RP62A was evaluated using a checkerboard approach as previously described (Ricciardelli et al. 2018). After biofilm formation and treatment, bacterial regrowth and biofilm biomass were assessed by absorbance at 600 nm, plating, and crystal violet staining to determine MBIC and MBEC values. MBIC was defined as the lowest concentration at which no visible biofilm formation was observed, while MBEC corresponded to the lowest concentration at which no regrowth occurred on agar and no residual biofilm biomass was detected. All experiments were performed in technical duplicates and independently repeated at least twice (biological duplicates).

### 2.13 Biocompatibility Assay

Immortalised human keratinocytes (HaCaT, Innoprot, Derio, Spain) and immortalised murine fibroblasts BALB/c‐3 T3 (ATCC, Virginia, USA) were maintained in Dulbecco's Modified Eagle Medium (DMEM) supplemented with 10% fetal bovine serum, 1% penicillin/streptomycin, and 2 mM L‐glutamine, under a humidified atmosphere containing 5% CO_2_ at 37°C. To assess the impact of *Ps*yOmp38 on cell viability, HaCaT cells were plated in 96‐well plates at a density of 2 × 10^3^ cells per well, and BALB/c‐3 T3 cells at 3 × 10^3^ cells per well. Twenty‐four hours post‐seeding, the cells were treated with increasing concentrations of the protein (ranging from 0.1 to 200 μg/mL) for 24, 48, and 72 h. After the treatment period, cell viability was assessed using the MTT assay (3‐(4,5‐dimethylthiazol‐2‐yl)‐2,5‐diphenyltetrazolium bromide), as previously described (Imbimbo et al. 2024). Cell viability was expressed as the percentage of living cells in the presence of the protein relative to control cells (calculated as the average between untreated cells and those exposed to the highest concentration of buffer). The protein was tested in three separate experiments, each performed in triplicate.

### 2.14 Emulsification Index Assay

The emulsification index (E_24_) was determined as previously reported (Blesic et al. 2018) with minor modifications. Briefly, by mixing 2 mL of Dectol (a mixture of decane and toluene in a 65:35 *v*/*v* ratio) with 1 mL of Omp38 proteins, prepared at different concentrations (30 μg/mL, 100 μg/mL, 500 μg/mL, 1 mg/mL) in 5 mL glass vials. The mixtures were vortexed at maximum speed using an ArgoLab MIX for 5 min and left undisturbed for 24 h. The E_24_ value was calculated as the ratio of the height of the emulsified layer to the total height of the liquid column, multiplied by 100.

### 2.15 Contact Angle Measurements

Advancing type contact angles with ultrapure water on PDMS, with and without the anti‐biofilm coatings, were measured with a locally manufactured contour monitor using the sessile drop (Van Oss et al. 1988) technique. For each assay, at least three droplets were deposited at different positions, and images were acquired 40 s after deposition. Measurements were performed on three independently prepared samples.

### 2.16 Preparation of the Antibiofilm PDMS Coatings and Activity Analysis in the Flow Cell System

The impact of *Psy*Omp38 protein coatings on *S. epidermidis* RP62A biofilm formation was assessed in a flow cell system (Stovall Life Science, USA), as previously described (Galdiero et al. 2021) with modifications. PDMS surfaces were coated with 250 μg of *Psy*Omp38 (approx. 26 μg/cm^2^), air‐dried under sterile conditions, and UV‐sterilised. Bacterial suspensions (OD_600_ = 0.2 in PBS + 2% BHI) were circulated at 170 μL/min using a peristaltic pump. After a 2.5 h adhesion phase and PBS washing, biofilms were grown for 24 h in 50% BHI/PBS. Finally, biofilms on coated and control surfaces were stained with the Filmtracer LIVE/DEAD kit (Invitrogen, USA) and analysed by confocal laser scanning microscopy (CSLM).

### 2.17 Statistics and Reproducibility of Results

Statistical evaluations were carried out using either a two‐tailed Student's *t*‐test or a two‐way ANOVA, followed by Tukey's post hoc test for multiple comparisons. A *p* ≤ 0.05 was considered to indicate statistical significance. All experiments were conducted in a minimum of three independent replicates, and the data are expressed as the mean ± standard deviation (SD). Analyses were performed using GraphPad Prism software (version 8; GraphPad Software Inc., La Jolla, CA, USA).

### 2.18 Bioinformatic Analysis

*Psy*Omp38 homologues present in the PDB, UniProt, and NCBI protein databases were identified by using the BLASTP server available at the NCBI site (https://blast.ncbi.nlm.nih.gov/Blast.cgi). Multiple alignments were performed by using Clustal Omega [30] visualised and optimised by using the Jalview software (Madeira et al. 2024; Waterhouse et al. 2009). Neighbour‐joining trees were obtained from the multiple alignments by using Jalview and visualised by TreeViewer 2.2.0 (https://treeviewer.org/). AlphaFold2 models were either obtained from the AlphaFold Protein Structure Database (https://alphafold.ebi.ac.uk/) or prepared by the ColabFold v1.5.5 server (https://colab.research.google.com/github/sokrypton/ColabFold/blob/main/AlphaFold2.ipynb).

## 3 Results

### 3.1 Production and Purification of Omp38+SP and Omp38‐SP Recombinant Proteins

**FIGURE 1:**
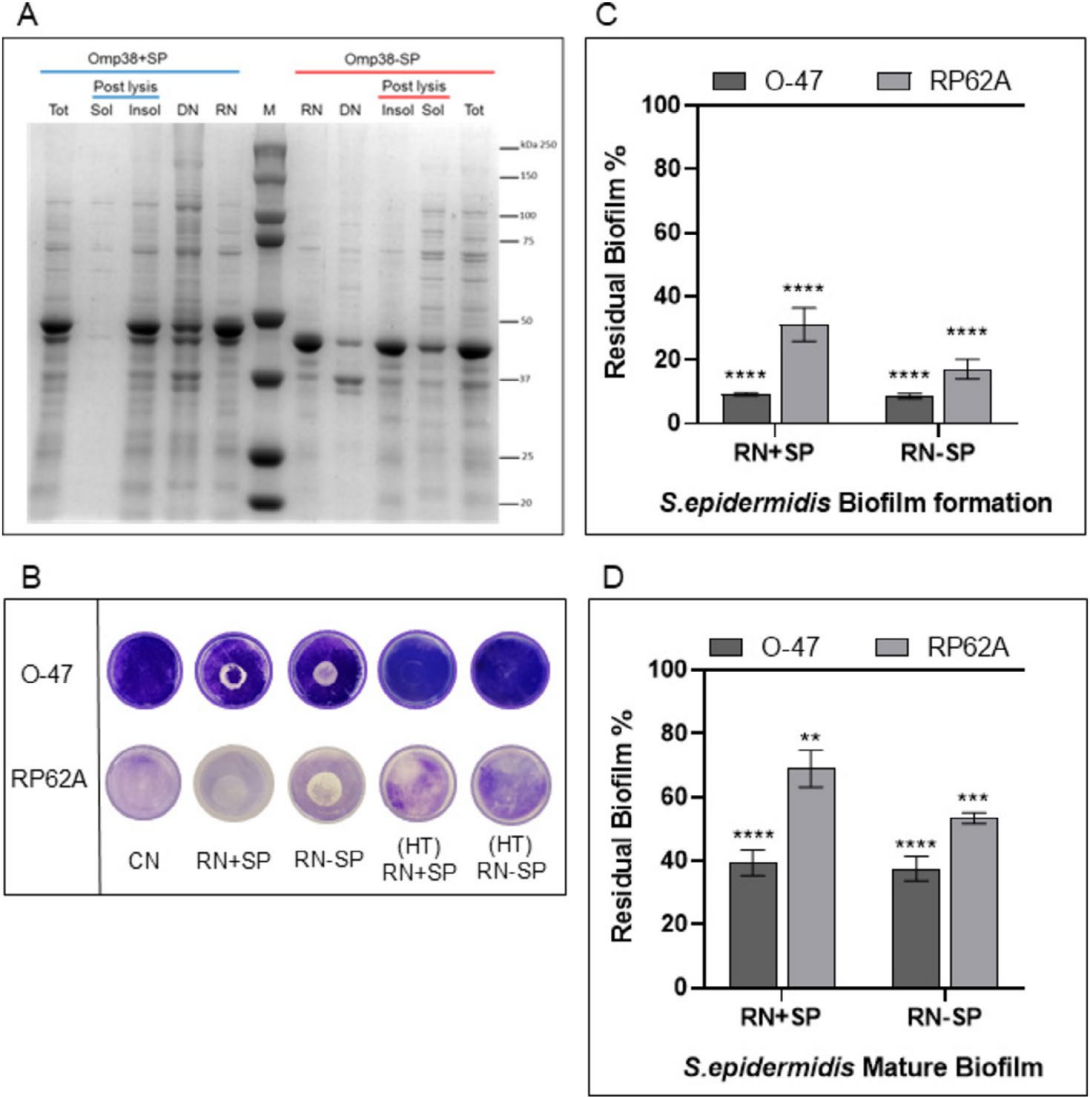
Production, recovery from inclusion bodies, and antibiofilm activities of Omp38 proteins. (A) SDS‐PAGE (10%) analysis of Omp38+SP (blue) and Omp38‐SP (red) expression and recovery from inclusion bodies. The gel shows *E. coli* BL21 (DE3) total extract (Tot) after induction, along with the soluble (Sol) and insoluble (Insol) protein fractions. Denatured (DN) and renatured (RN) protein fractions were obtained by solubilisation in 8M urea. M: molecular weight marker. (B) Biofilm formation by *S. epidermidis* RP62A and O‐47 strains in 24‐well polystyrene plates coated with either 50 mM TrisHCl pH 9.0 (CN, control) or renatured protein fractions (RN + SP, RN − SP). As control RN proteins were heat‐denatured at 90°C for 20 min, named respectively (HT) RN + SP, (HT) RN − SP. (C) Inhibition of initial *S. epidermidis* biofilm formation by renatured protein fractions. (D) Effect of renatured protein fractions on preformed 24 h mature biofilms. In (C) and (D), data are presented as mean ± SD from six independent replicates and expressed as a percentage of biofilm relative to the untreated control (100%). Biofilm formation was considered unaffected in the 90%–100% range. Statistical significance was assessed using Student's *t*‐test or two‐way ANOVA with Tukey's post hoc test; *p* < 0.05 (**p < 0.01, ***p < 0.001, ****p < 0.0001).

The native protein *Psy*Omp38 has a signal peptide for translocation across the inner membrane. Therefore, two recombinant protein versions were produced: one with the signal peptide, designated Omp38+SP, and one without it, named Omp38‐SP. As described in the M&M section, *Psy*Omp38 genes were cloned into the pET28a(+) vector under the control of an inducible lac operon using *E. coli* BL21(DE3) or *E. coli* C41(DE3) as a host for the heterologous production. The production was initially carried out in both *E. coli* strains, but as the expression in *E. coli* C41(DE3) was not effective (data not shown), the production continued using *E. coli* BL21(DE3). Production was optimized by testing different temperatures and induction conditions (Figure S1). Optimal expression of Omp38+SP was achieved by inducing with 1 mM IPTG at 20°C, followed by 24 h of incubation. For Omp38‐SP, optimal induction was obtained with 2 mM IPTG, and protein production was carried out for 6 h at 25°C. Both proteins were successfully produced, and after cell lysis, the soluble and insoluble protein fractions were investigated by SDS‐PAGE, revealing that both proteins were mainly found in the insoluble fraction (Figure 1A). Initial recovery attempts of proteins from inclusion bodies were performed using non‐denaturing methods (Klausser et al. 2023) with detergents like CHAPS, DDM, OG, and LDAO (data not shown). These solvents allowed partial solubilization of inclusion body aggregates, though with low yield. Therefore, a denaturation‐renaturation protocol, previously used to solubilize recombinant outer membrane protein A (OmpA) of *A. baumannii* expressed in *E. coli* (McConnell and Pachón 2011), was applied for both proteins. Protein recovery and solubility were assessed by SDS‐PAGE (Figure 1A). Given the anti‐adhesive properties of the CATASAN complex, the renatured fractions were tested for their ability to impair the initial attachment of *S. epidermidis* strains to the polystyrene surface, by coating assay (Figure 1B). Both renatured fractions altered the surface properties, reducing the initial attachment of *S. epidermidis* O‐47 and *S. epidermidis* RP62A strains. Furthermore, both the renatured fractions proved able to reduce the biofilm development and promote the detachment of staphylococci mature biofilm (Figure 1C,D).

**FIGURE 2:**
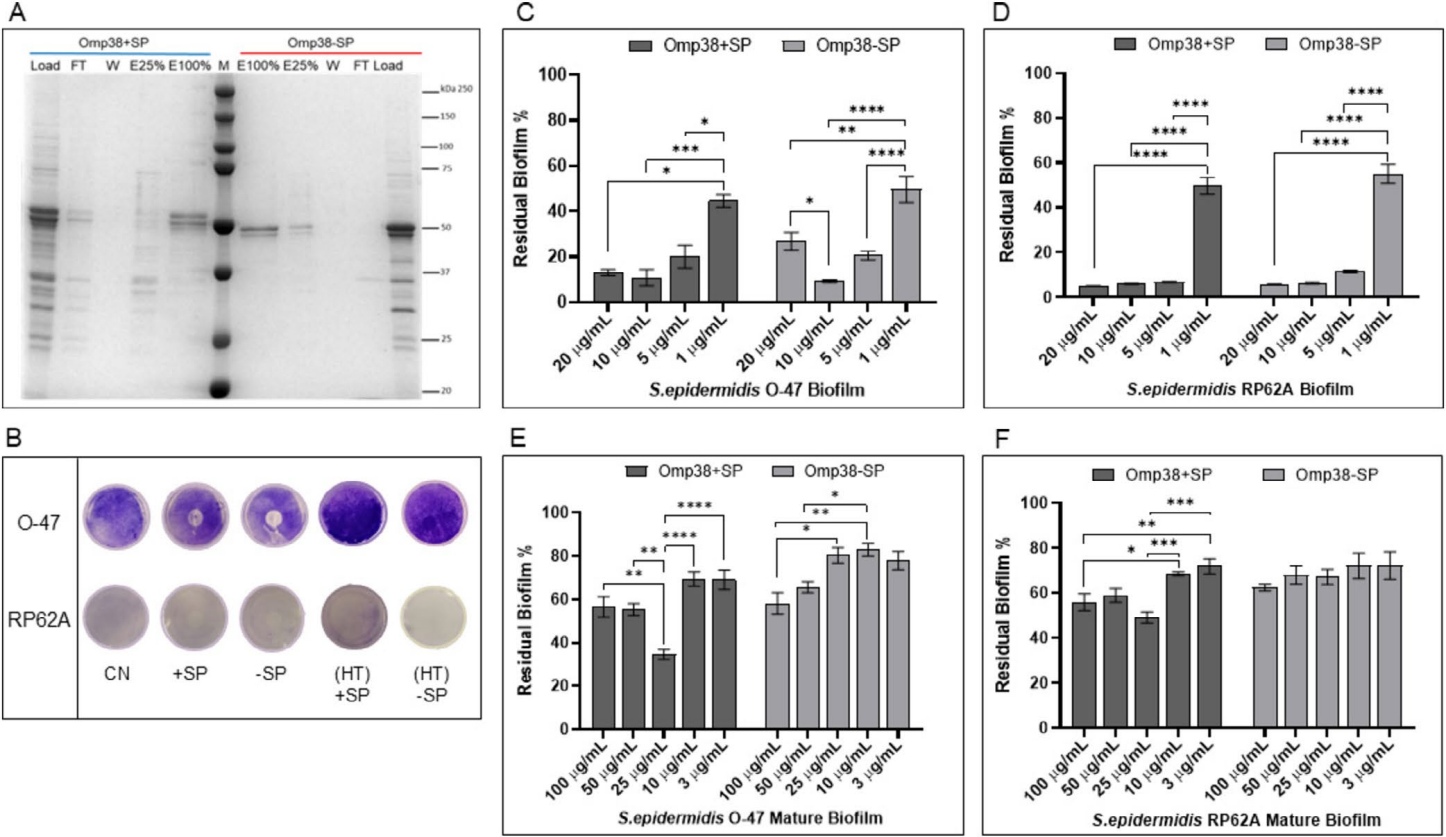
Purification and antibiofilm activities of Omp38 proteins. (A) SDS‐PAGE (10%) analysis of Omp38+SP (blue) and Omp38‐SP (red) purified via anion exchange chromatography. The gel shows the renatured protein fractions loaded onto the column (Load), the flow‐through (FT), the wash (W), and elution fractions obtained with increasing NaCl concentrations (E25%: 50 mM TrisHCl +250 mM NaCl; E100%: 50 mM TrisHCl +1 M NaCl). M: molecular weight marker. (B) Biofilm formation by *S. epidermidis* RP62A and O‐47 in 24‐well polystyrene plates coated with 50 mM TrisHCl pH 9.0 (CN, control) or purified Omp38 proteins (Omp38‐SP and Omp38+SP) at 10 μg/mL. As the control purified proteins were heat‐denatured at 90°C for 20 min, they were named (HT) + SP and (HT) − SP, respectively. (C, D) Antibiofilm activity of purified Omp38 proteins at various concentrations (20, 10, 5, 1 μg/mL) against *S. epidermidis* O‐47 (panel C) and RP62A (panel D) biofilm formation. (E, F) Effect of purified proteins at different concentrations (100, 50, 25, 10, and 3 μg/mL) on 24 h preformed *S. epidermidis* O‐47 (panel E) and RP62A (panel F) mature biofilms. In panels (C–F), data are shown as mean ± SD from six independent replicates. Results are expressed as the percentage of biofilm biomass relative to the untreated control (set to 100%). Biofilm formation was considered unaffected in the 90%–100% range. All treatments with Omp38 proteins resulted in a statistically significant reduction of biofilm compared to the untreated control (*p* < 0.05 or lower). The statistical treatments indicated in the graphs refer to comparisons between protein concentrations within the same treatment group. Statistical significance was assessed using Student's *t*‐test or two‐way ANOVA with Tukey's post hoc test (*p < 0.05, **p < 0.01, ***p < 0.001, ****p < 0.0001).

Anion Exchange Chromatography was performed to purify the proteins from the renatured fractions. SDS‐PAGE analysis of the fractions (Figure 2A) revealed that both proteins were eluted mainly at 100% NaCl and that both proteins displayed a double band on the gel with about a 3 kDa difference between the two bands (Image Lab tool analysis). Further analyses were conducted to determine the nature of the double band. The studies were conducted on Omp38‐SP; however, identical results were obtained for the protein containing a signal peptide. Specifically, mass spectrometry results (Figure S2B) confirmed that both bands on the SDS‐PAGE corresponded to the recombinant Omp38–SP protein. Additionally, UREA‐PAGE analysis showed a single migrating band (Figure S2A), indicating that the anomalous migration pattern arises from the specific conditions of SDS‐PAGE. This hypothesis is supported by literature data, which indicate that outer membrane proteins similar to Omp38 stably bind SDS, and that this binding is responsible for the electrophoretic mobility shift (Rath et al. 2009).

### 3.2 Omp38+SP and Omp38‐SP Antibiofilm Activities

The Omp38+SP and Omp38‐SP up to a concentration of 210 μg/mL were found to be unable to interfere with the planktonic growth of *S. epidermidis* (data not shown). The antiadhesive and biofilm‐inhibiting activities of purified recombinant proteins were evaluated. The ability of purified Omp38+SP and Omp38‐SP to impair *S. epidermidis* strains surface adhesion was assessed by coating assay (Figure 2B), while their ability to prevent staphylococcal biofilm formation was evaluated by adding purified proteins to the medium at the beginning of sessile growth and reported in Figure 2C,D where the biofilm obtained in the absence of the proteins is reported as 100%. The activity against mature biofilms was determined by adding the purified proteins after biofilm formation (Figure 2E,F). Results demonstrated that both recombinant proteins reduced the initial attachment of *S. epidermidis* O‐47 and *S. epidermidis* RP62A, interfered with biofilm development, and promoted detachment of mature *S. epidermidis* biofilms. Notably, both proteins were effective at very low concentrations, as inhibition of biofilm formation was observed starting from 1 μg/mL, and the effect on mature biofilm was achieved from 3 μg/mL. It is interesting to note that the activity of the protein on biofilm formation is dose‐dependent, whereas its effect on disrupting the mature biofilm does not appear to be strictly dose‐dependent.

**FIGURE 3:**
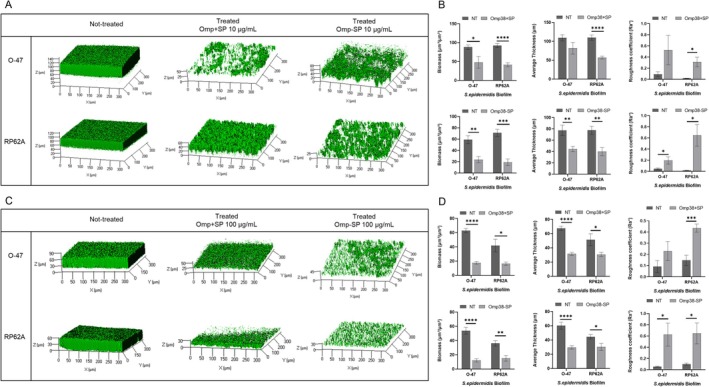
Confocal analysis of S. epidermidis biofilms treated with purified Omp38 proteins. (A) CLSM images of *S. epidermidis* RP62A and O‐47 biofilms formed after 24 h of growth in wells coated with either culture medium (NT), Omp38–SP, or Omp38 + SP (10 μg/mL). Biofilms were stained with the LIVE/DEAD Biofilm Viability Kit. (B) Quantitative analysis using COMSTAT software of biomass, average thickness, and roughness of the biofilms shown in (A). Data are expressed as the percentage relative to the untreated control (NT, set at 100%). Each value represents the mean ± SD of two independent biological replicates. Biofilm formation was considered unaffected within the 90%–100% range. (C) CLSM images of mature 24 h biofilms treated with purified proteins (100 μg/mL) for an additional 24 h, stained with the LIVE/DEAD Biofilm Viability Kit. (D) COMSTAT analysis of biomass, average thickness, and roughness of biofilms is shown in (C). Data represent the mean ± SD of two independent replicates and are expressed as the percentage of biofilm relative to the untreated control (100%). Statistical significance in panels (B) and (D) was determined using Student's *t*‐test; *p* < 0.05 (*p < 0.05, **p < 0.01, ***p < 0.001, ****p < 0.0001).

The antibiofilm activity of pure proteins on the biofilm structure was evaluated by Confocal Laser Scanning Microscopy (CLSM) by the LIVE/DEAD Biofilm Viability Kit. As shown in Figure 3, CLMS analysis has proven that the proteins can reduce *S. epidermidis* biofilm formation without influencing cell viability and promote the detachment of staphylococci mature biofilm (Figure 3A,C). The CLSM stack images were analysed using the COMSTAT image analysis software package (Heydorn et al. 2000) to assess structural biofilm parameters. As expected, the biomass and average thickness of the biofilm were lower in the presence of proteins compared to the untreated samples. Additionally, in the treated sample, a rise in the roughness coefficient was observed (Figure 3B,D). This latter factor is dimensionless and quantifies variations in biofilm thickness; it is commonly used as an indirect indicator of biofilm heterogeneity. The analysis demonstrated that the treatment led to a more unstructured biofilm.

Since the two proteins exhibited comparable antibiofilm properties, both in terms of anti‐adhesive activity, inhibition, and disruption of the mature biofilm, subsequent analyses were carried out exclusively with the protein Omp38–SP, hereafter referred to as *Psy*Omp38.

Moreover, given the emulsifying ability of the CATASAN complex, this property was evaluated for the proteins analysed in the present study. The emulsification assay demonstrated that both proteins were capable of stabilising emulsions (Figure S3A–B); however, their emulsifying efficiency was lower compared to that of CATASAN (D'Angelo et al. 2022). Notably, emulsions were stable for over 1 month, in a concentration‐dependent manner (Figure S3C).

### 3.3 Effect of *Psy*Omp38 on Eukaryotic Cell Viability

**FIGURE 4:**
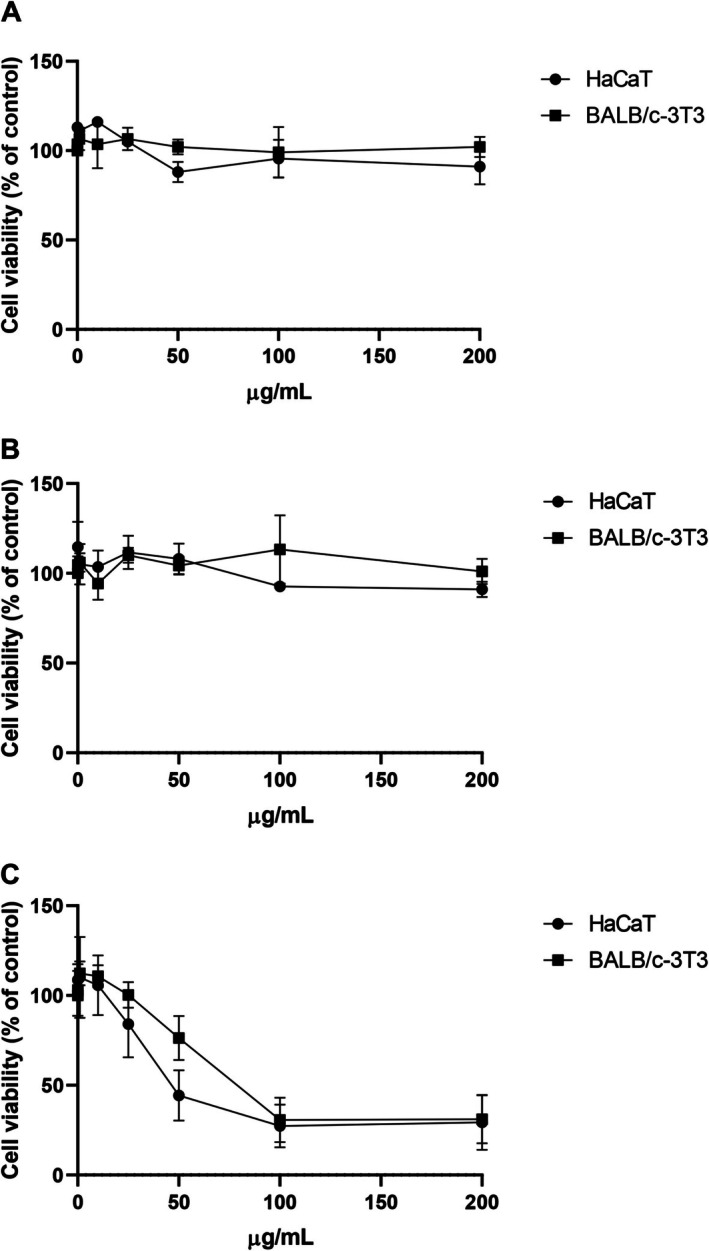
Effect of PsyOmp38 on immortalised cell viability. Effect of *Psy*Omp38 on immortalised cell viability. Dose–response curve of HaCaT cells (black circles) and BALB/c‐3T3 (black squares) upon incubation with increasing concentration of protein (0.1–200 μg/mL) for 24 h (A), 48 h (B) and 72 h (C). Cell viability was assessed by the MTT assay and expressed as percentage of treated cells with respect to control cells. Control cells are represented by the average between untreated cells and cells supplemented with the highest concentration of buffer. Values are given as means ± SD (*n* ≥ 3).

To further investigate a possible medical application of *Psy*Omp38, its biocompatibility was evaluated on two immortalised eukaryotic cell lines, BALB/c‐3T3 and HaCaT cells, as they represent the outermost layer of the skin and the dermis, respectively. Cells were incubated with rising protein concentrations and for different lengths of time, and the results are reported in Figure 4. After 24 and 48 h incubation, no toxicity was observed on any of the cell lines studied at all the concentrations tested (Figure 4A,B). After 72 h incubation (Figure 4C), *Psy*Omp38 exerted a cytotoxic effect on both cell lines, showing typical dose–response curves. The toxic effect observed after 72 h incubation differed for the two cell lines analysed, as the IC50 values (i.e., the protein concentration able to reduce cell survival by 50%) were 42 ± 3 μg/mL for HaCaT cells and 81 ± 4 μg/mL for BALB/c‐3T3 cells.

### 3.4 Use of *Psy*Omp38 in Combination With Vancomycin

The combination of *Psy*Omp38 with antibiotics was tested on mature biofilms. Specifically, these experiments used vancomycin as the antibiotic and *S. epidermidis* RP62A as the target strain. In order to rule out any potential interference between the *Psy*Omp38 and vancomycin, the Minimum Inhibitory Concentration (MIC) of vancomycin on *S. epidermidis* RP62A was determined both in the presence and absence of *Psy*Omp38 (up to 250 μg/mL). The MIC value was assessed following CLSI guidelines (Weinstein 2019). In all tested conditions, vancomycin's efficacy was unaffected by the presence of *Psy*Omp38 (data not shown).

The Minimum Biofilm Eradication Concentration (MBEC) was used to estimate the antimicrobial activity of vancomycin against mature biofilms (Castaneda et al. 2016). The possible cooperative effect of *Psy*Omp38 and vancomycin was assessed on 24 h mature biofilm, and the synergy was evaluated using the checkerboard method, as described in the M&M section. The results shown in Table 1 demonstrated that in the presence of 100 μg/mL of the protein, the concentration of vancomycin necessary to eradicate the biofilm is reduced fourfold, and in the case of a concentration below this value, the required vancomycin concentration is halved.

**TABLE 1:** Synergistic activity of Omp38 protein and vancomycin against *S. epidermidis* RP62A. Minimum biofilm inhibitory concentration (MBIC) and minimum biofilm eradication concentration (MBEC) values were determined for *Psy*Omp38 in combination with vancomycin. Synergy was evaluated based on a reduction in MBIC or MBEC values in the presence of combined treatment compared to vancomycin alone. Data are expressed as concentrations (μg/mL) obtained from at least two independent biological replicates.

	Vancomycin	Vancomycin + *Psy*Omp38 100 μg/mL	Vancomycin + *Psy*Omp38 50 μg/mL	Vancomycin + *Psy*Omp38 25 μg/mL	Vancomycin + *Psy*Omp38 12.5 μg/mL	Vancomycin + *Psy*Omp38 6.25 μg/mL	Vancomycin + *Psy*Omp38 3.125 μg/mL
MBIC	60	15	15	30	30	30	30
MBEC	60	15	15	30	30	30	30

### 3.5 Preparation of the *Psy*Omp38 PDMS Coating and Antibiofilm Activity Evaluation in Flow Cells

To create an antibiofilm coating for medical devices able to prevent *S. epidermidis* infections, a surface of the silicone‐based polymers, polydimethylsiloxane (PDMS) (Wolf et al. 2018), as modified by the adsorption of *Psy*Omp38 on the PDMS surface. The coating was achieved by physical adsorption of the antibiofilm protein onto the PDMS surface using the drop‐casting method. A solution of *Psy*Omp38 of 200 μg/mL in 50 mM TrisHCl, pH 9.0, was placed dropwise on the PDMS surface, and the coating was achieved after evaporation. The derivatised surface was washed seven times with water to evaluate whether the washings were able to remove the protein from the PDMS surface. The variation of the Water Contact Angle (WCA) was used to evaluate whether the protein was able to modify the surface properties and to detect the presence of the protein on the PDMS surface even after washing.

**FIGURE 5:**
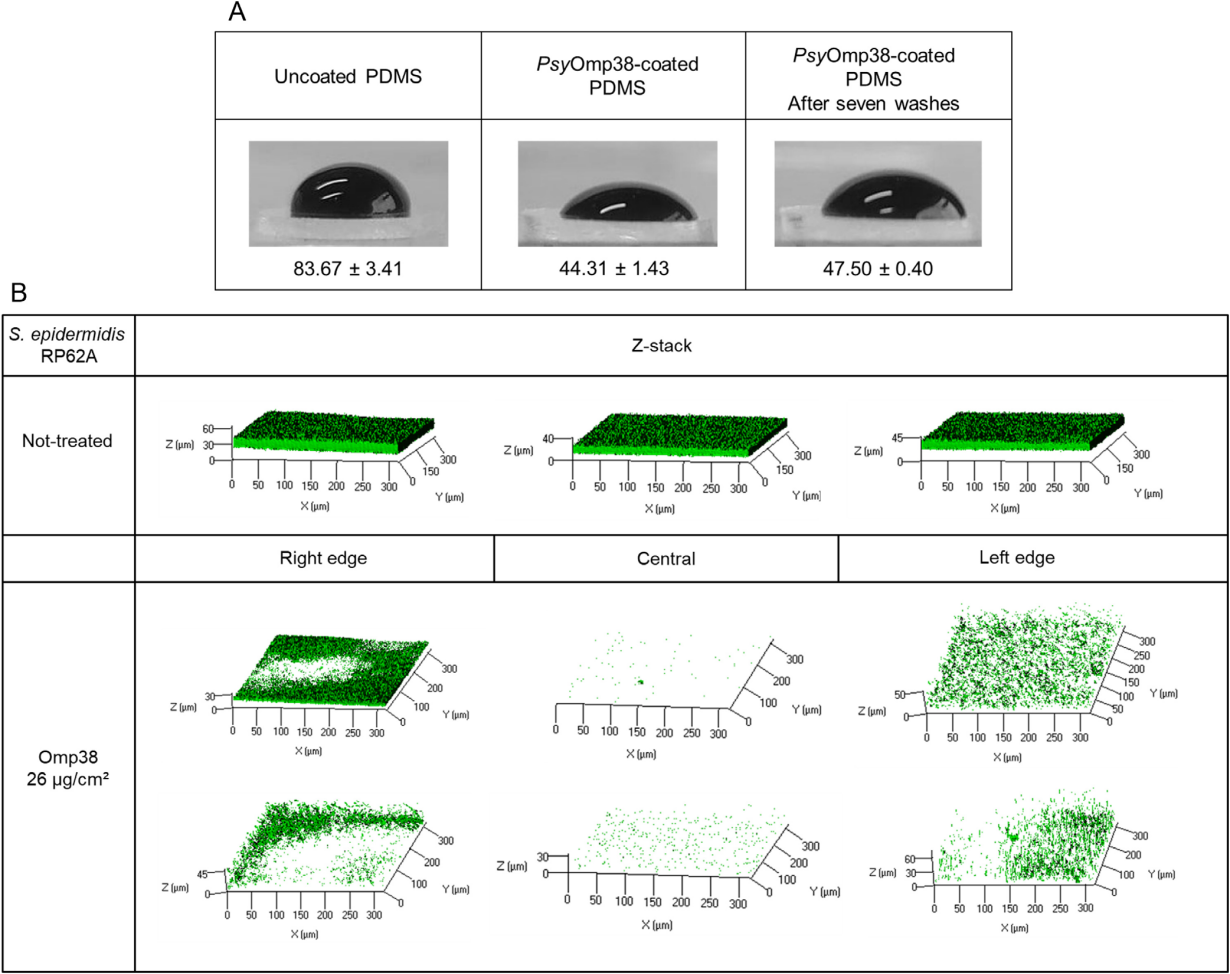
Effect of *Psy*Omp38 on PDMS hydrophobicity and activity analysis on S. epidermidis RP62A biofilm formation under flow conditions. (A) Representative images of water droplets and corresponding contact angle measurements on uncoated PDMS, PDMS coated with *Psy*Omp38, and PDMS coated with *Psy*Omp38 after seven washes with water. Each data point represents the mean ± SD of three independent measurements. (B) Confocal laser scanning microscopy (CLSM) analysis of *S. epidermidis* RP62A biofilms formed on PDMS surfaces after 24 h of growth under continuous flow. PDMS surfaces were coated with either buffer (50 mM TrisHCl pH 9.0, NT), *Psy*Omp38 (surface concentration: 26 μg/cm^2^), and subsequently inoculated with *S. epidermidis* RP62A under continuous flow for 24 h. Biofilms were stained using the LIVE/DEAD Biofilm Viability Kit and imaged at three distinct positions along the flow channel (right side, center, and left side) to assess spatial distribution. The experiment was performed in two independent biological replicates. These images highlight the impact of protein‐coated surfaces on biofilm development under shear stress.

WCA measurements of the PDMS surface with and without the *Psy*Omp38 coating (Figure 5A) revealed that the contact angle of unmodified PDMS was about 80 degrees, whereas after the protein adsorption, the contact angle was about 45 degrees. Once it was demonstrated that it is possible to create a protein‐derivatised PDMS, the antibiofilm efficacy of this material was evaluated in a flow system. *S. epidermidis* RP62A biofilm formation on PDMS, with and without *Psy*Omp38 coatings, was evaluated by a convertible flow cell. Briefly, *S. epidermidis* cells were allowed to grow for 24 h in the convertible flow cells, which were connected in parallel to the flow system, one containing uncoated PDMS and the other PDMS coated with *Psy*Omp38. *S. epidermidis* RP62A biofilm was then analysed using CLSM (Figure 5B). CLSM investigation confirmed the capability of the coating to reduce the biofilm formation of *S. epidermidis* RP62A. Although derivatisation effectively decreases *S. epidermidis* biofilm formation on the PDMS surface, the effect appears to be non‐uniform. Certain areas still showed residual biofilm formation, although reduced. This irregularity is likely due to uneven surface coverage resulting from the coating strategy employed; the coating by casting may have led to heterogeneous deposition of the protein across the surface.

### 3.6 Bioinformatic Analysis of *Psy*Omp38

**FIGURE 6:**
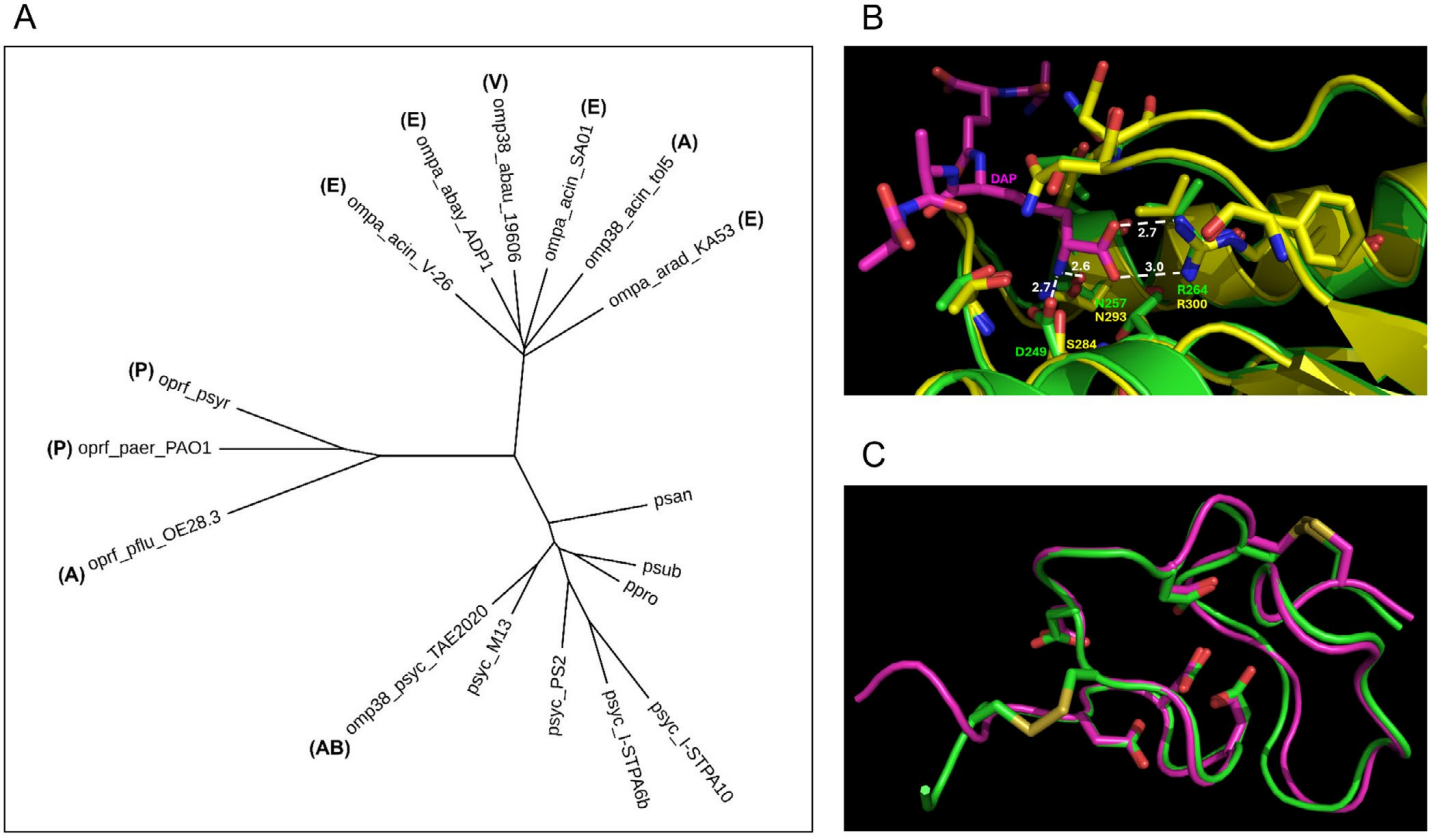
Bioinformatic analysis of *Psy*Omp38. (A) Neighbour‐joining tree derived from the multiple alignment shown in Figure S1. The letters in bold indicate the function of the protein when known: A, adhesion; AB, antibiofilm; E, emulsifier; P, porin/osmotic stress response; V, virulence factor. (B) Comparison between the crystallographic structure of the periplasmic domain of *A. baumannii* Omp38 in complex with a peptidoglycan fragment (PDB code 3TD5) and the AlphaFold2 model of *Psy*Omp38 periplasmic domain. Green cartoon, *A. baumannii* Omp38; yellow cartoon, *Psy*Omp38. The residues of the peptidoglycan binding pocket and the peptidoglycan fragment are shown as sticks. Sticks are coloured by atom type: nitrogen, blue; oxygen, red; carbon of *A. baumannii* Omp38, green; carbon of the peptidoglycan fragment, magenta; carbon of *Psy*Omp38, yellow. Hydrogen bonds formed by the diaminopimelate (DAP) side chain are shown as a white dashed line. Distances are in Å. (C) Comparison between the AlphaFold2 models of the conserved portion of the linker regions from *P. aeruginosa* PAO1 OprF and *Psy*Omp38. Green cartoon, OprF; magenta cartoon, *Psy*Omp38. Side chains of cysteine and conserved aspartate residues are shown as sticks. Sticks are coloured by atom type: nitrogen, blue; oxygen, red; sulfur, yellow; carbon of OprF, green; carbon of *Psy*Omp38, magenta.

*Psy*Omp38 belongs to a wide and heterogeneous family of outer membrane proteins that includes, among others, OmpA‐like proteins, Omp38‐like proteins, and the outer membrane porins F (OprF). These proteins share an architecture characterised by a *N*‐terminal transmembrane β‐barrel domain with porin function and a C‐terminal periplasmic domain with a diaminopimelate binding site that mediates binding to peptidoglycan. A linker of variable length, likely unstructured, connects the two domains. Recently few proteins of this family have been characterised: the OmpA‐like proteins from *A. radioresistens* strains KA53 (Toren et al. 2001), S13 (Violetta et al. 2014), *A. baylyi* ADP1 (Walzer et al. 2006), *Acinetobacter* sp. SA01 (Shahryari et al. 2021) and *Acinetobacter* sp. V‐26 (Mujumdar et al. 2019) are secreted emulsifiers; Omp38 from *A. baumannii* strains ATCC 19606 and ATCC 17978 (Lucaßen et al. 2021) are virulence factors able to induce apoptosis in human cell lines; Omp38 from *Acinetobacter* sp. Tol 5 (Ishikawa et al. 2016) is essential for the correct display of an autotransporter adhesin; OprF proteins from *P. aeruginosa* PAO1 (Paulsson et al. 2021) and *P. syringae pv. Syringa* (Cody and Gross 1987) have porin activity and controls cell shape and stability in low‐osmolarity media, whereas OprF from *P. fluorescens* OE 28.3 is a root adhesin (De Mot et al. 1992). These proteins were used to prepare a multiple alignment with *Psy*Omp38 and seven further uncharacterised Opm38‐like proteins from different *Psychrobacter* strains, with a percentage of identity with *Psy*Omp38 between 60% and 90% (Figure S4). The seven *Psychrobacter* Omp38 proteins were arbitrarily selected among about 120 Omp38 proteins from *Psychrobacter* strains present in the NCBI protein database. The neighbour‐joining tree derived from the multiple alignment shows that the proteins from *Acinetobacter*, *Psychrobacter*, and *Pseudomonas* strains form three clearly distinct branches, irrespective of the function attributed to the proteins (Figure 6A). *Psy*Omp38 and the other *Psychrobacter* Omp38‐like proteins share some sequence traits in common with the *Acinetobacter* Omps and others with the *Pseudomonas* OprF porins, as well as unique traits. The transmembrane barrel domain is moderately conserved, with the most variable regions corresponding to the extracellular loops of the barrel (Figure S4).

The first strand‐loop‐strand motif is the least conserved, with the loop region particularly variable not only among the three groups but also within each single branch (Figure S4). Toren and colleagues identified four hydrophobic regions of the barrel domain that are necessary for the emulsifying activity of the proteins from strains KA53, S13, ADP1, and V‐26 (Toren et al. 2002); (Walzer et al. 2006, 2009). These regions are very well conserved in all the *Acinetobacter* sequences, partially conserved in the *Psychrobacter* sequences, where some substitutions increase hydrophilicity (Figure S4), and not conserved in the *Pseudomonas* OprF porins (Figure S4). The periplasmic peptidoglycan‐binding domain is better conserved. In particular, the residues that form the diaminopimelate binding pocket are mostly well conserved (Figure S4). An interesting exception is one of the three residues that directly bind the side chain of the diaminopimelate residue, that is, an arginine residue that forms two hydrogen bonds with the carboxylate group of the diaminopimelate and an aspartate and an asparagine residue that form hydrogen bonds with the amino group of diaminopimelate (Figure 6B). In the *Psychrobacter* Omp38‐like proteins, the aspartate is substituted by a serine residue (Figure S4). The comparison between the crystallographic structure of the periplasmic domain of the protein from *A. baumannii* in complex with a peptidoglycan fragment (PDB code 3TD5) and the AlphaFold2 model of *Psy*Omp38 shows that the architecture of the diaminopimelate binding pocket is well conserved and that the peptidoglycan fragment could still be accommodated in the pocket of *Psy*Omp38 from a steric point of view, however, the impact of the substitutions on the affinity is not easily predictable by an *in silico* analysis. The linker between the two domains is the most variable region of the protein family. Intriguingly, the linker of the *Psychrobacter* proteins shares several similarities to the linker of the OprF porins from *P. aeruginosa* PAO1 and *P. syringae* , in particular the presence of an acidic region with regularly spaced aspartate residues (containing DXD, DXXD, and DXXXD motifs, frequently found in divalent cation binding sites) (Sissi and Palumbo 2009; Mazumder et al. 2023) and two conserved cysteine residues present in the conserved motifs CP and GCP (Figure S4). Figure 6C shows the comparison of AlphaFold2 models of the conserved portion of the linker regions from *P. syringiae*, OprF and *Psy*Omp38. The backbone and the conserved disulfide are completely superimposable; moreover, the conserved aspartate residues form a cluster in agreement with the hypothesis that these residues could be involved in the binding of metal cations.

## 4 Discussion

CATASAN, being a complex composed of the *Psy*Omp38 protein, LPS, and a high‐molecular‐weight polysaccharide, likely of capsular origin (unpublished results), is intrinsically difficult to study, both structurally and functionally. A reductionist approach aimed at the individual structural and functional characterisation of the various components of the complex may seem simplistic, but it is nonetheless effective. Investigating the role of each molecule involved in the complex separately could allow for the evaluation of the individual contribution of each component to the overall activity of CATASAN. This approach also lays the foundation for understanding the structure/function relationships underlying the complex's mechanism of action. Moreover, the detailed characterisation of the individual components is essential for exploring their potential biotechnological applications. In the case of investigating the role of the *Psy*Omp38 protein, the most logical approach to uncover its characteristics was its recombinant production in *E. coli* cells. The initial idea was to produce two forms of the protein: one containing a signal peptide and the other lacking this signal for periplasmic translocation. This approach aimed at a comparison between the protein version targeted to the periplasm and the one expressed in the cytoplasm to assess which of the two would yield a higher amount of soluble protein. Both forms were produced as cytoplasmatic inclusion bodies, a very common occurrence when expressing outer membrane integral proteins (McConnell and Pachón 2011). It was nevertheless decided to purify both versions to evaluate any potential effect of the signal peptide on the protein's functionality. The presence of this sequence of 22 amino acids, characterised by a charged N‐region followed by a hydrophobic H‐region, was found to influence only the emulsifying capacity of the protein, which, when equipped with this sequence, appears to exhibit improved emulsifying properties (Figure S3). Both proteins, refolded from inclusion bodies and purified, were tested against the various stages of *S. epidermidis* biofilm formation. The proteins demonstrated CATASAN‐like activity, preventing cell adhesion to polystyrene, inhibiting biofilm formation, and disrupting established biofilms. These activities were exerted by the proteins even at low concentrations. However, a quantitative comparison with CATASAN is challenging. CATASAN is a complex mixture with an undefined molecular weight and unknown stoichiometric ratios among its components, making it impossible to directly compare its activity to that of the purified proteins at equivalent concentrations. Whether and how the LPS or the polysaccharide contributes to these activities remains to be further investigated. Nevertheless, the strong antibiofilm properties of the protein make it a promising tool for combating biofilm‐associated *S. epidermidis* infections. Therefore, its biocompatibility was evaluated on HaCaT and BALB/c‐3T3 cells, as they represent the skin, in which keratinocytes are the outermost layer that covers the fibroblasts layer, a target for *S. epidermidis* infections. Data demonstrated that the protein is fully biocompatible with all the cell lines analysed up to 48 h. However, a cytotoxic effect was observed upon 72 h, but the concentrations at which a toxic effect is observed are higher than those at which the protein is active; therefore, the possibility of using the protein in combination with a standard antibiotic for the treatment of established infections was explored. The use of the antibiofilm protein allowed for up to a fourfold reduction in the amount of vancomycin required to achieve biofilm eradication, lowering the MBEC value. This property of the protein makes its use promising in combination with traditional antibiotics for the treatment of infections caused by *S. epidermidis* . The idea of combining the treatment of classical antibiotics with antibiofilm molecules has been reported in several studies (Belfield et al. 2017; Li et al. 2024) and it has proven capable of reducing, even by tenfold, the concentration of antibiotics required to eradicate the infection (Li et al. 2024).

In the fight against biofilm‐dependent infections, it is important to eradicate established infections, but it is even more crucial to prevent their formation, especially on medical devices. Indeed, the challenge of surface modification of medical devices to prevent bacterial adhesion and biofilm formation has become an essential aspect for medical implants (Li et al. 2023). To prevent biofilm formation, we investigated the potential of converting PDMS into an antibiofilm material through protein adsorption. After confirming the stability of *Psy*Omp38 adsorption under the tested conditions, the antibiofilm activity of the functionalized surface was evaluated under flow conditions to better mimic real‐life environments. In this case, the results demonstrated the good potential of the proposed approach, showing that the presence of the protein reduces biofilm formation on the silicone surface. However, the data also highlighted the limitations of the derivatisation technique employed. Indeed, the casting‐based coating method resulted in a surface with non‐uniform antibiofilm activity. Future efforts will certainly focus on developing a more uniform and homogeneous coating.

In parallel with the functional characterisation of the protein, preliminary structural studies on the protein were conducted. Structural analysis of the protein revealed that *Psy*Omp38 belongs to a broad family of outer membrane proteins characterised by an N‐terminal transmembrane β‐barrel domain, a C‐terminal periplasmic domain responsible for peptidoglycan binding, and a linker region connecting the two domains. Only a few members of this family have been characterised to date, including some OmpA‐like proteins from *Acinetobacter* (Walzer et al. 2006), Omp38 from *A. baumannii* (McConnell and Pachón 2011), Omp38 from *Acinetobacter* sp. Tol 5 (Ishikawa et al. 2016), and the OprF protein from *P. aeruginosa* (Paulsson et al. 2021). These known proteins were aligned with *Psy*Omp38 and seven uncharacterised Omp38‐like proteins from *Psychrobacter* strains.

Phylogenetic analysis grouped the proteins into three main clades: *Acinetobacter*, *Psychrobacter*, and *Pseudomonas*. Multiple sequence alignment revealed that *Psy*Omp38 and other *Psychrobacter* Omp38‐like proteins share some sequence traits in common with *Acinetobacter* Omps and others with the *Pseudomonas* OprF porins, as well as unique traits. The transmembrane β‐barrel domain is moderately conserved across the family; hydrophobic regions likely involved in the emulsifying activity are well conserved in *Acinetobacter*, partially conserved in *Psychrobacter*, and absent in *Pseudomonas*. The periplasmic peptidoglycan‐binding domain is broadly conserved, particularly at residues known to bind diaminopimelate, although *Psychrobacter* Omp38‐like proteins display a key aspartate‐to‐serine substitution at this site. The linker connecting the two domains is the most variable region, and in *Psychrobacte*r Omp38‐like proteins, it shares notable similarities with the linker found in OprF porins from *P. aeruginosa* PAO1 and *P. syringae* . Taken together, these findings indicate that *Psychrobacter* Omp38‐like proteins represent a distinct subfamily within this broader group. To the best of our knowledge, the data presented in this study are the first functional insights reported for a member of this newly identified protein family.

The structural features that emerge from the analysis of *Psy*Omp38 three‐dimensional structure provide some insights into the understanding of the protein's properties. Notably, the presence in the barrel domain and the other hydrophobic regions present in the protein could explain the emulsifying properties of the protein, these properties are shared with other similar *Acinetobacter* outer membrane (Navon‐Venezia et al. 1995; Toren et al. 2001; Walzer et al. 2006; Shahryari et al. 2021).

In the case of anti‐biofilm properties, it is not easy to establish a structure–function correlation, but some hypotheses can be cautiously proposed. The ability of the protein to adhere to polystyrene and PDMS could be associated with the N‐terminal region, which, by exposing hydrophobic amino acids on the outer surface of the barrel domain, may promote this type of interaction. The protein adhesion lowers the surface hydrophobicity, and this decrease can reduce *S. epidermidis* adhesion to polystyrene (Cerca et al. 2005). While surface hydrophobicity is known to influence initial bacterial attachment (Krzywicka et al. 2022) it is unlikely to be the single factor. Importantly, initial attachment and biofilm formation are distinct steps governed by different molecular mechanisms, and strong initial adhesion does not necessarily result in robust biofilm development (Cerca et al. 2005). Therefore, the change in surface hydrophobicity may justify the inhibition of bacterial adhesion to the surface, but it does not justify the inhibition of biofilm formation or the disruption of mature biofilm. *Psy*Omp38's ability to inhibit biofilm formation and promote detachment of mature biofilms may be linked to its interference with the structure and stability of the *S. epidermidis* biofilm matrix, this ability could be linked to its emulsifying properties. Amphipathic molecules with surfactant activity are known to exhibit antibiofilm effects (e Silva et al. 2017). In the case of *S. epidermidis*, for example, phenol‐soluble modulins are involved in biofilm dispersion (Le et al. 2019). Assuming that the biofilm inhibition activity and the disintegration of the mature biofilm are at least partially attributable to the protein's emulsifying properties, it remains unclear why the former displays a clear concentration‐dependent effect, while the effect on the mature biofilm does not. Probably, in the case of the mature biofilm, the protein can only act on the outer layers of the matrix, and the lack of homogeneity in the reaction (the *Psy*Omp38 activity on biofilm) could explain why the effect is not strictly dose‐dependent. Although the data obtained so far may suggest some differences in *Psy*Omp38 action in the case of biofilm inhibition compared to mature biofilm disruption, in both cases, the resulting biofilm matrix is structurally different from that of an untreated biofilm. Indeed, CLSM analysis of protein‐treated biofilms shows not only a reduction in biomass but also significant structural changes in the biofilm architecture. Future studies will aim to clarify the mechanism of action of *Psy*Omp38 on *S. epidermidis* biofilm.

## 5 Conclusions

The data presented demonstrate that *Psy*Omp38 can inhibit cell adhesion, prevent biofilm formation, and promote the disaggregation of preformed *S. epidermidis* biofilms. Moreover, the studies support the potential therapeutic use of the protein, in combination with conventional antibiotics, for the eradication of established infections, as well as its application in the development of antibiofilm materials aimed at preventing the onset of biofilm‐associated infections. Additionally, the structural studies on *Psy*Omp38 not only introduce a new family of outer membrane proteins but also lay the groundwork for future research to elucidate the molecular mechanisms underlying the antibiofilm activity of this promising protein.

## Author Contributions

**Diana Olimpo:** writing – original draft, methodology, investigation, data curation. **Caterina D'Angelo:** writing – original draft, methodology, investigation, data curation. **Paola Imbimbo:** writing – review and editing, investigation, formal analysis. **Marco Morelli:** investigation, data curation. **Maria Luisa Tutino:** writing – review and editing, formal analysis. **Andrea Carpentieri:** writing – review and editing, investigation. **Daria Maria Monti:** writing – review and editing, investigation, formal analysis. **Eugenio Notomista:** writing – review and editing, investigation, formal analysis. **Ermenegilda Parrilli:** writing – original draft, conceptualisation, data curation, formal analysis.

## Conflicts of Interest

The authors declare no conflicts of interest.

## Supporting information


**Data S1:** mbt270249‐sup‐0001‐DataS1.docx.

## Data Availability

All data generated or analysed during this study are included in this published article and its Supporting Information files.
