# Supplementary material for: Novel Insights Into the Struggle Against Biofilm: The Psy Omp38 Protein From the Antarctic Marine Bacterium Psychrobacter sp. TAE2020
Source: Microb Biotechnol. 2025 Oct 7;18(10):e70249. doi: 10.1111/1751-7915.70249 (PMC12504631; doi:10.1111/1751-7915.70249)
Supplement: Supplementary file 1 — Data S1: mbt270249‐sup‐0001‐DataS1.docx. [file MBT2-18-e70249-s001.docx]

**Novel Insights into the Struggle Against Biofilm: The *Psy*Omp38 Protein from the**

**Antarctic Marine Bacterium *Psychrobacter* sp. TAE2020**

**Diana Olimpo, Caterina D’angelo, Paola Imbimbo, Marco Morelli, Maria Luisa Tutino, Andrea Carpentieri, Daria Maria Monti, Eugenio Notomista, Ermenegilda Parrilli**

**Table S1.** *List of bacterial strains, plasmids, and primers used in this study.*

| Strain | Description | Reference and/or source |
| --- | --- | --- |
| *Psychrobacter* sp*.* TAE2020 | Polar marine bacterium from Antarctic Sea water ^a^ | Liège collection |
| *Staphylococcus epidermidis* RP62A | Reference strain isolated from infected catheter | ATCC collection |
| *Staphylococcus epidermidis*  O-47 | Clinical isolate from septic arthritis | Heilmann C. et., 1996 |
| *Escherichia coli* TOP 10 | [supE44, ΔlacU169 (ϕ80 lacZΔM15) hsdR17, recA1, endA1, gyrA96, thi-1, relA1] | Lab stock |
| *Escherichia coli* BL21(DE3) | F^–^ ompT gal dcm lon hsdS_B_(r_B_^–^m_B_^–^) λ(DE3) [lacI lacUV5 T7p07 ind1 sam7 nin5]) [malB^+^]_K-12_(λ^S^) | Lab stock |
| *Escherichia coli* C41(DE3) | F^–^ ompT gal dcm hsdS_B_(r_B_^-^ m_B_^-^) (DE3) | Lab stock |
| Cell lines / RRID | **Description** | **Source** |
| HaCaT / CVCL_0038 | sex: male; Tissue of origin: Back, skin, epidermis; species: Human, Caucasian | Innoprot, 2015 |
| BALB/3T3 / CVCL_0184 | sex: unspecified; Tissue of origin: embryo; species: Mouse | ATCC, 2015 |
| Plasmid |  |  |
| pET28a | Commercial vector |  |
| Oligonucleotide | **Primer sequence** | |
| Omp38 – *Nco*I – SP – fw | 5’-CGT**CCATGG**GAAAATTGAATAAAATTGC-3’ | |
| Omp38 – *BamH*I – rv | 5’-GCT**GGATCC**TTAGTTGATTACCATATC-3’ | |
| Omp38 – *Nco*I – fw | 5’-ATA**CCATGG**GTGTTACTATCAGTCC-3’ | |


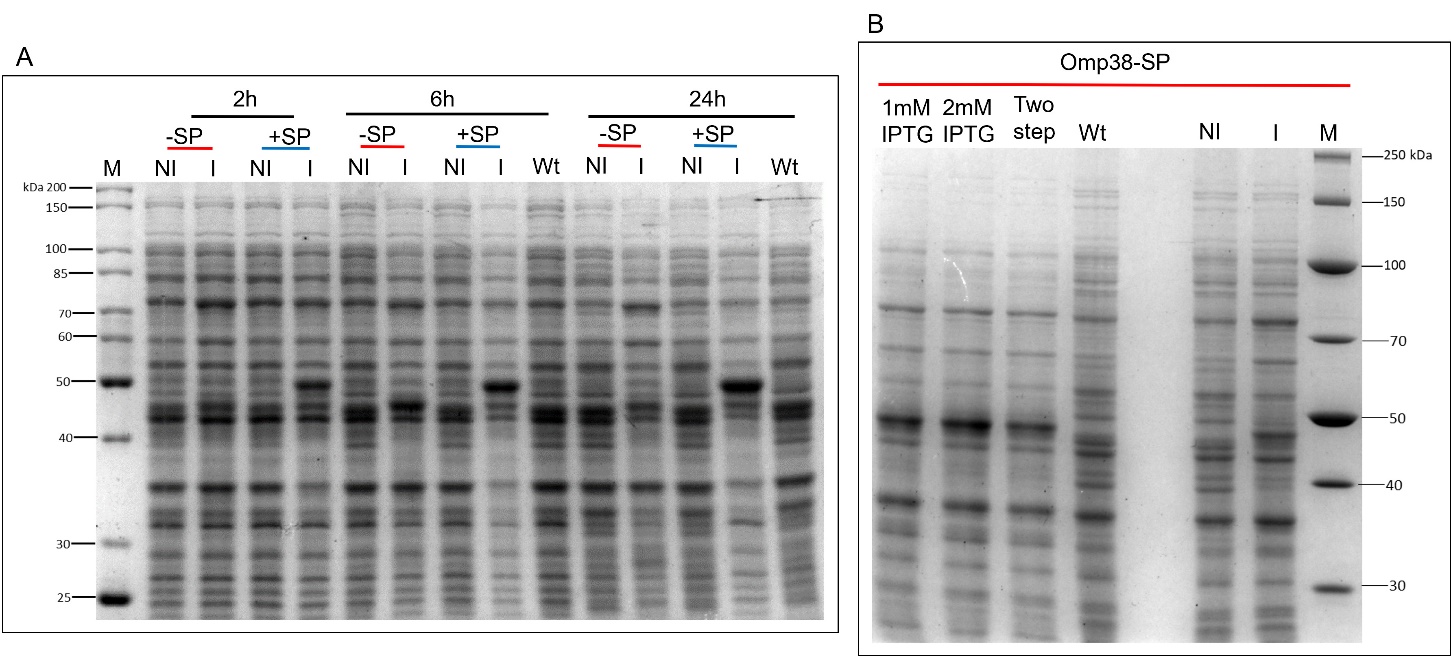


**Figure S1.** *Production conditions optimization for Omp38 proteins*

**(A)** SDS-PAGE (10%) cell extract of recombinant production of Omp38 in *E. coli* BL21(DE3), induction with 1 mM IPTG. Analysis of fractions collected at different times from the induction at 2 h, 6 h, and 24 h. The analysis was performed on the extracts of the induced (I) and not induced (NI) fractions of the production of the recombinant protein in the presence (+SP) and absence(-SP) of the secretory signal peptide. For clarity, the red color represents the analyses performed on the Omp38-SP, while the blue color represents Omp38+SP. The wild type is present as a control (Wt). M: molecular weight marker **(B)** SDS-PAGE (10%) of different inducer concentrations (1mM, 2mM, and 2mM in two steps) for Omp38–SP production. The wild type (Wt) and the non-induced (NI) fractions are present as controls. M: molecular weight marker.


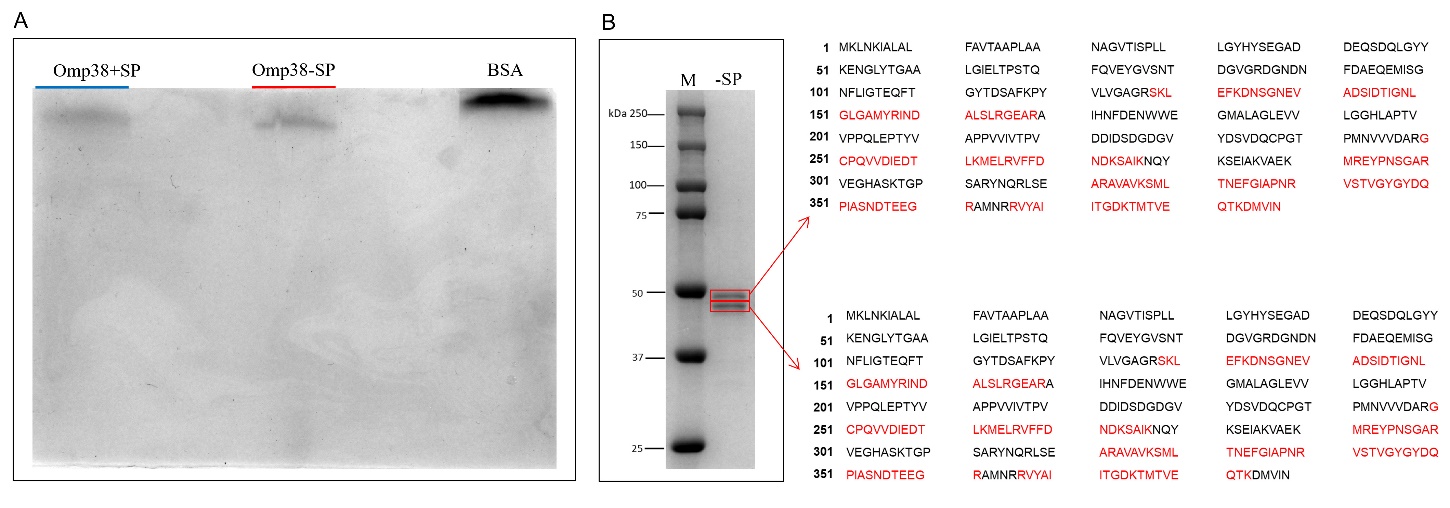


**Figure S2.** *Urea-PAGE and Mass Spectrometry reveal identity of double band as single protein species*

**(A)** Urea-PAGE gel in the presence of Omp38 proteins and BSA as standard. **(B)** SDS-PAGE (10%) of purified Omp38-SP used for mass spectrometry.


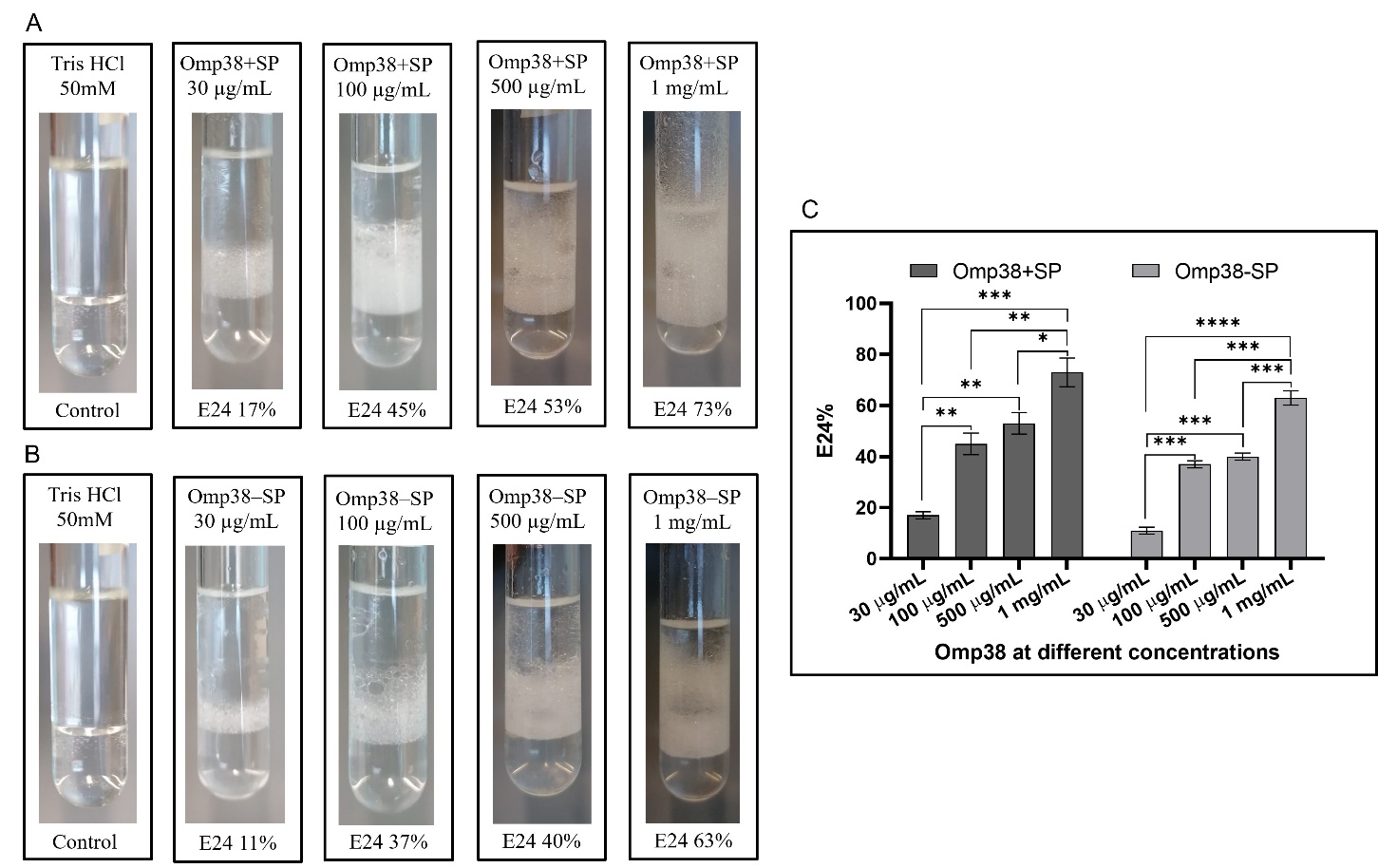


**Figure S3.** *Emulsifying activity of Omp38 proteins.*

Representative images **(A-B)** and quantification **(C)** of the emulsification index obtained after 24 h (E24%) with increasing concentrations (30, 100, 500, 1000 µg/mL) of Omp38+SP **(A)** and Omp38-SP **(B)** and 50 mM Tris HCl pH 9.0 (control). The emulsification index was calculated as the ratio between the emulsion layer's height and the liquid column's total height, expressed as a percentage. Data are shown as mean ± SD from two independent experiments. All treatments with Omp38 proteins resulted in a statistically significant reduction of biofilm compared to the untreated control (p < 0.05 or lower); therefore, these comparisons are not shown in the graphs. The statistical differences indicated in the graphs refer exclusively to comparisons between protein concentrations within the same treatment group, highlighting concentration-dependent effects. Statistical significance was assessed using Student’s *t*-test or two-way ANOVA with Tukey’s post hoc test (^*^*p* < 0.05, ^**^*p* < 0.01, ^***^*p* < 0.001, ^****^*p* < 0.0001).


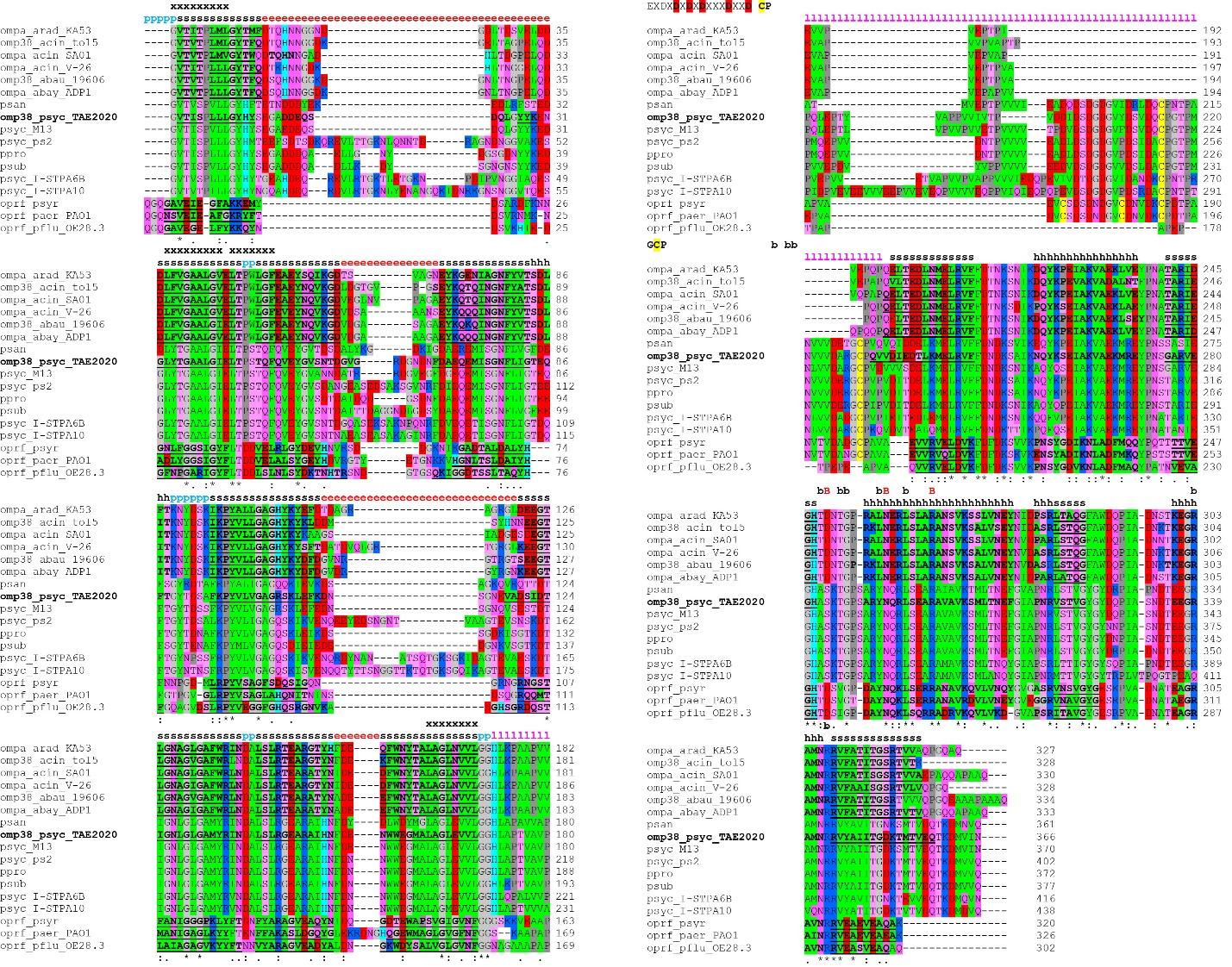


**Figure S4.** *Multiple sequence alignment of selected PsyOmp38 homologues.*

Residues are colored by properties: hydrophobic (WFLIMVYA), green; basic (KR), blue; acidic (DE), red; hydrophobic, pink (ST), magenta (NQ); proline, dark grey; glycine, light grey; histidine, cyan; cysteine, yellow. Bold, alpha helix; bold/underlined, beta strand. Consensus secondary structure, orientation of the loops of the barrel domain, and notable sequences are shown above the alignment: **h**, alpha helix; **s**, beta strand, **p**, periplasmic loop; **e**, extracellular loop; **l**, interdoman linker; **x**, sequences essential for the emulsifying activity in OmpA-like proteins from *Acinetobacter* *radioresistens* strains KA53 and S13, *Acinetobacter* *baylyi* ADP1, *Acinetobacter* sp. SA01 and *Acinetobacter* sp. V-26; **b**, residues of the diaminopimelate binding pocket; **B**, residues that directly bind the side chain of diaminopimelate.

Protein name abbreviations:

ompa_arad_KA53: *Acinetobacter radioresistens* KA53 OmpA-like protein (Alasan) (NCBI db, AY033946)

omp38_acin_tol5 : *Acinetobacter* sp. Tol 5 OmpA-like protein (UniProtKB, A0A160PAH3)

ompa_acin_SA01: *Acinetobacter* sp. Strain SA01 OmpA-like protein (NCBI db, WP_166170865)

ompa_acin_V-26 : *Acinetobacter* sp. V-26 OmpA-like protein (UniProtKB, Q4FCM8)

omp38_abau_19606: *Acinetobacter baumannii* ATCC 19606 Omp38 (UniProtKB, Q6RYW5)

ompa_abay_ADP1: *Acinetobacter baylyi* ADP1 OmpA-like protein (UniProtKB, Q6FE98)

psan: *Psychrobacter* *sanguinis* (NCBI db, WP_312750340.1)

omp38_psyc_TAE2020: *Psychrobacter* sp. TAE2020 *PsyOmp38*

psyc_M13: *Psychrobacter* sp. M13 Omp38 (NCBI db, WP_305899547.1)

psyc_ps2: *Psychrobacter* sp. Ps2 Omp38 (NCBI db, WP_238051689.1)

ppro: *Psychrobacter* *proteolyticus* Omp38 (NCBI db, WP_114701177.1)

psub: *Psychrobacter* *submarinus* Omp38 (NCBI db, WP_201610930.1)

psyc_I-STPA6B: *Psychrobacter* sp. I-STPA6b Omp38 (NCBI db, WP_227429455.1)

psyc_I-STPA10: *Psychrobacter* sp. I-STPA10 Omp38 (NCBI db, WP_230660697.1)

oprf_psyr: *Pseudomonas* *syringae* pv. *syringae* Outer membrane porin F (UniProtKB, P22263)

oprf_paer_PAO1: *Pseudomonas* *aeruginosa* PAO1 Outer membrane porin F (UniProtKB, P13794)

oprf_pflu_OE28.3: *Pseudomonas* *fluorescens* OE 28.3 Outer membrane porin F (UniProtKB, P37726)
